# Perceived stress of mental demands at work, objective stress and resilience – an analysis of the LIFE-Adult-study

**DOI:** 10.1186/s12995-023-00388-0

**Published:** 2023-09-07

**Authors:** Franziska U. Jung, Alexander Pabst, Francisca S. Rodriguez, Melanie Luppa, Christoph Engel, Toralf Kirsten, Veronica Witte, Nigar Reyes, Markus Loeffler, Arno Villringer, Steffi G. Riedel-Heller

**Affiliations:** 1 Institute of Social Medicine, Occupational Health and Public Health (ISAP), Faculty of Medicine, Leipzig University, Ph.-Rosenthal-Str. 55, 04103 Leipzig, Germany; 2 RG Psychosocial Epidemiology & Public Health, Greifswald, Germany; 3 LIFE - Leipzig Research Centre for Civilization Diseases, Leipzig University, Leipzig, Germany; 4 Institute for Medical Informatics, Statistics and Epidemiology, Leipzig University, Leipzig, Germany; 5 Clinic of Cognitive Neurology, University of Leipzig Medical Center, Leipzig, Germany; 6 Department of Neurology, Max Planck Institute for Human Cognitive and Brain Sciences, Leipzig, Germany

**Keywords:** Mental demands, Chronic stress, Work overload, Work discontent

## Abstract

**Background:**

So far, previous research suggests positive effects of mental demands at the workplace. However, it may depend on how stressfull these demands are perceived on an individual level.

**Objective:**

The aim was to build on previous research by investigating how mental demands are related to stress, overload, and work discontent and whether this relationship is mediated by individuals resources, such as resilience.

**Method:**

A sub-sample of the LIFE Adult Cohort (n = 480) was asked to answer questions on sociodemographic characteristics, objective stress (using the Trier Inventory of Chronic Stress (TICS)), and perceptions of stress with regard to verbal and executive mental demands at work.

**Results:**

According to generalized linear regression models, higher verbal as well as executive mental demands were associated with higher levels of chronic stress, work overload and discontent. Higher levels of resilience were associated with lower levels of these outcomes. Analyses regarding interaction effects revealed that the interaction between resilience and perceived stress of verbal mental demands was significant only in terms of work overload.

**Conclusion:**

Higher perceived stressfulness of mental demands was associated with higher chronic stress, work overload and work discontent. Therefore, mental demands should be targeted by occupational interventions that aim to improve job conditions and employees‘ overall well-being. Besides resilience, other potential influencers or personal resources should be focused on in future studies to develop interventions.

## Introduction

A job or a “good” employment can be an essential resource for mental health, as unemployment has been associated with mental and physical health issues [1, 2]. At the same time, short- as well as long-term psychological distress at the workplace can lead to negative consequences and negatively impact overall well-being. Especially within the last decade, occupational environments and job demands have changed due to innovations, digitalization and demographic transitions. Apart from many benefits associated with these changes - such as reducing risk of error or efficiency enhancement – new work environments and practices may also lead to increases in occupational demands, workload and distress.

### Mental demands at work – are they beneficial or harmful?

Occupational activities often involve different requirements that may have negative or beneficial effects on mental health. According to the Job-Demand-Control-Model by Karasek [3], overall stress at work may be harmful for mental health – depending on the amount of job control that is experienced by the individual. The Job-Demand-Control-Model proposes that the two fundamental aspects of occupations—“psychological job demands” and “job decision latitude”—are the main causes of workplace-related stress. Karasek defines psychological job demands, as psychological stressors prevalent in the workplace (e.g. mentally demanding work). The word “job decision latitude” captures one’s ability to control activities or skill application [4]. Several studies currently found evidence for the impact of modern work environments and demands on mental well-being [5, 6]. Specifically, mental demands have been associated with wide-reaching benefits, as it may buffer against age-related cognitive decline and delay the onset of dementia [7–11]. So far, the literature available with regard to the consequences for mental health is not yet sufficiently covered. One study investigated the connection between mental demands and mental health found that two factors – the possibility to control working time and the potential for learning and improvement – may for instance reduce the severity of depressive symptoms [12]. Their results are based on a specific sample aged 50 years and older and the question remains whether this may also apply to younger employees. Besides, they also found mediation effects of perceived fit between person and job, arguing for an employee-driven process. In other words, the relationship between mental demands and possible consequences may not only be individual but also depend on how well the employees‘ skills match their jobs‘ requirements (i.e. whether tasks involve doing unleared things) and how an individual is coping with a certain demand.

Using a multi-dimensional approach, it has been suggested to assess mental demands at work based on a theory-driven categorization described by Then and colleagues [11, 13, 14]. To sum up their findings, certain occupational context indices exist, that are relevant in determining the characteristics of an enriched environment at work, providing enhanced sensory as well as cognitive stimulation. Initially, they developed four different indices: novelty, fluid, verbal and executive. Based on further investigations using these concept, work environment characterized by high verbal as well as executive proficiencies, may be of great relevance to preserve better cognitive functioning [9–11, 13]. Examples for executive mental demands include characteristics such as *developing objectives or strategies* or *resolving conflicts*, whereas verbal mental demands emcompass demands such as *getting information* or *providing consultation and advice*.

### Perceived stressfullness of mental demands and individual resources

The simple existence of a mental requirement does not have to mean an associated burden. The nature of the stressor and the situation in which it occurs (i.e. the interaction between the individual and their environment) are also important, as suggested by the transactional stress model [15–17]. In this context, previous study results also indicate that subjectivity, i.e. individual assessment and perception and the degree of the stressor, also plays a major role [18]. In other words, how stressful one perceives a mental demand may play a more determining role. A study looking at the connection between mental demands at work and stress and burnout confirmed that it is not the requirement per se, but the extent to which something is perceived as stressful [19]. In this study, only some mental demands were perceived as stressful, when their levels was also high. Further analyses revealed that only higher stress was associated with burnout symptoms, but not higher levels of mental demands, therefore actual levels may not be as relevant as (individual) appraisal of stressful demands. The question arises whether increases in burnout symptoms may be related to increased chronic stress and workload, and also impact job discontent. In this context, the stress buffering model assumes that specific psychosocial parameters may be protective against stress [15, 20]. The strength of the consequences associated with (work-related) stress may rather depend or be mediated by personal characteristics. Self-efficacy refers to the individuals‘ ability to cope with challenging situations, for instance by mediating the relationship between stress and depression [21]. A related construct, which has also been associated with reduced stress in the literature to date, is resilience, known as the ability to adjust to difficulties that pose a threat to ones‘ survival, development, or functioning [22]. The concept of resilience has been used to explain why some employees are less impacted by stressful events than others [23]. This was further evaluated by another study, showing that resilience could act as a buffer to lessen the negative consequences of work stress by moderating the relationship between stress and burnout [24].

### Aims and hypotheses of the current study

We spend a lot of our lives in our profession, so it is of great importance to examine the consequences associated with employment in more detail in order to enable good and healthy work on the one hand and to shape future employment in a sustainable manner. Therefore, the aim of this study is to build on these results and, using a larger sample, to further analyze to what extent differences in the perception of high mental professional demands promote work-related chronic stress, overload and dissatisfaction and negative effects can possibly be buffered by the presence of pronounced resilience. In a first step, we aimed to replicate and elaborate on previous results [19], investigate the association between verbal and executive mental demands and stress across different occupational groups. However, instead of focusing on burnout, we investigated the link between mental demands and occupational well-being in general (i.e. chronic stress, work overload and job discontent), as these constructs may be accompanied by negative effects on overall mental health, as well as absentism, reduced productivity, high fluctuation or even early retirement. We hypothesize that higher perceived stress may be associated with higher levels of chronic stress (H1), work overload (H2) and discontent (H3). In a second step, we investigated the mediating role of resilience on these associations in order to derive a ground for workplace interventions. In this context, we hypothesize that resilience may have a positive (hence buffering) effect on perceived stress of mental demands and moderate the relationship between mental demands and occupational well-being (H4).

## Method

### Data collection

Data were derived from the Follow-up LIFE-Adults-Study of the Leipzig Research Centre for Civilization (LIFE), containing a population-based representative sample of people living in Leipzig (Eastern Germany). The LIFE baseline sample was randomly drawn from population registers (between 2011 and 2014) and contains residents with ages ranging between 18 and 80, however, as stated in the study protocol, the main focus was on subjects aged 40 to 80, and participants 39 of age and younger were also included but underrepresented. The first follow-up assessment took place between 2017 and 2021.

Overall, participants in the study received a series of assessment procedures at the study center, including the gathering of their sociodemographic data, medical history, details regarding lifestyle factors, and other medical examinations. The sole conditions for exclusion were pregnancy and a lack of proficiency in German. Further information on the study procedure as well as data collection and ethical considerations have been described elsewhere in greater detail [25]. The LIFE-Adult-Study was approved by the institutional ethics board of the Medical Faculty of the University of Leipzig. Written informed consent was obtained from all participants.

**Fig. 1:**
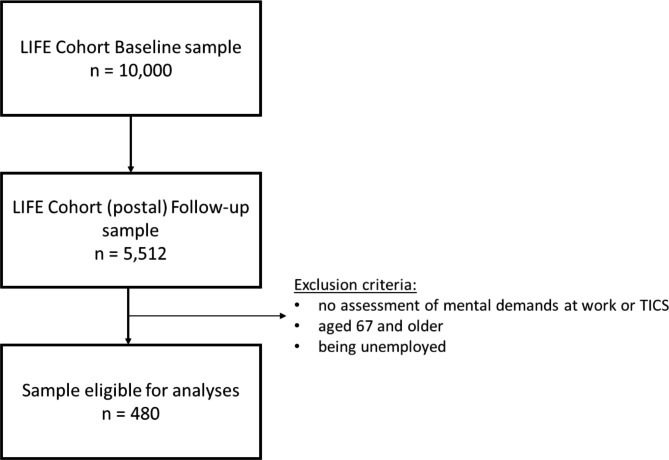
Overview of the sample under investigation

Two study variables, perceived stressfulness of mental demands as well as chronic stress (measured by Trier Inventory of Chromic Stress, TICS), were only included within follow-up assessments. Therefore, the current analyses is based on the follow-up data. The analytical sample was restricted by excluding people who were either unemployed or retired, or who were older than 67 years (retirement age in Germany). Therefore, the overall sample that was suitable for final analyses consisted of 480 participants (see Fig. 1).

### Instruments

Sociodemographic characteristics included information on age, gender, marital status and education. The latter was converted into low, middle and high educational level according to the CASMIN categorization scheme [26]. Occupational information was dichotomized depending on whether participants currently work full-time or part-time. In addition, chronic mental health was also assessed, revealing that n = 23 participants have been diagnosed with anxiety disorder and n = 42 participants have been diagnosed with depression.

Mental demands at the workplace were assessed based on the definition of Enriched Environment at Work [11, 19]. Participants were asked to rate the perceived stress associated with twelve occupational mental demands on a five-point-scale (1 = no stress, 5 = high stress). These demands were divided into two indices: verbal (example demands: getting information, providing consultation and advice) and executive mental demands (example demands: developing objectives and strategies, scheduling work and activities).

Work-related stress was assessed using the Trier Inventory of Chronic Stress (TICS), which has been shown to have good psychometric properties [27, 28]. The scale consists of 57 items that can be subdivided into nine area-specific domains. For the purpose of the current study, only three subscales were used (Work Overload, Chronic Stress and Work Discontent). Example items included: “I have too many tasks to perform.“ (Work Overload); “Times when none of my tasks seem meaningful to me.“ (Work Discontent) and “Times when I worry a lot and cannot stop.“ (Chronic Stress).

In addition, resilience was measured using the German version of the Resilience Scale, RS-11 [29], containing eleven items that need to be answered using a 7-point-Likert-scale (1 = strongly disagree; 7 = strongly agree). Again, this scale has been shown to have good psychometric properties [30].

### Statistical analysis

Descriptive statistics are shown as means with standard deviations (quantitative) or number of cases with percentages (qualitative). A sensitivity analysis was performed, with differences between selected and excluded participants being tested using Mann Whitney U and Chi^2^ (χ^2^)-tests. Associations between mental demands, resilience and stress were examined using multivariate regression analyses. Three models were evaluated, distinguishing between the three work-related stress domains as dependent variables. Due to the skewed distribution of the work-related variables, generalized linear regression models (GLM) were calculated, applying a gamma distribution and a log link function. First, the models included variables for perceived stress related to verbal (1) and executive mental demands (2) as well as resilience (3) as unconditional effects on work-related stress (main effects models). Because of different ranges regarding their scores, these three variables were z-standardized in order to make them comparable. Second, we estimated conditional effects by adding interaction terms between mental demand domains and resilience to test whether the effect of perceived stress related to mental demands on work-related stress was moderated by resilience (interaction effects models). All models were adjusted for age, gender, education, income, and occupation. All data were weighed for age, gender and level of education to be representative of the German general population in 2016 (see Tables 1 and 2). StataSE Version 16 has been used for all statistical analyses.

**Table 1:** Descriptive analysis of sociodemographics, work-related stress, and resilience

		Sample under investigation (n = 480)
**Sociodemographic characteristics**	**Age** (range: 26–66)	49.1 (SD: 9.3)
	**Gender**	
	male female	276 (57.5%) 204 (42.5%)
	**Income** (household), €^1^	
	< 2000 2000–3000 3000–4000 4000–5000 > 5000 refused to answer	16 (3.2%) 59 (12.3%) 127 (26.5%) 82 (17.1%) 103 (21.5%) 93 (19.4%)
	**Education** (CASMIN)	
	low/middle^1^ high	285 (59.4%) 195 (40.6%)
	**Occupation**	
	Full-time Part-time	410 (85.4%) 70 (14.6%)
**Assessments**	**Trier Inventory of Chronic Stress (TICS)** ^2^	
	**Chronic Stress**, range: 0–42 **Work Overload**, range: 0–19 **Work Discontent**, range: 0–27	12.1 (SD: 7.6) 4.2 (SD: 3.3) 8.2 (SD: 4.8)
	**Resilience**, range: 12–77^3^	60.9 (SD: 10.0)

Note: ^1^less than 2% were categorized as “low”; ^2^ higher values indicating higher stress/overload/discontent; ^3^ higher values indicating greater resilience

**Table 2:** Ratings of perceived stress related to verbal and executive mental demands

Mental Demands		Perceived Stress
**Verbal**	1. Providing Consultation and Advice	2.4 (SD: 1.1)
	2. Getting Information	2.3 (SD: 1.0)
	3. Evaluating Information	2.3 (SD: 1.0)
	4. Interpreting the Meaning of Information	2.4 (SD: 1.0)
	5. Updating and Using Relevant Information	2.4 (SD: 1.1)
	6. Analyzing Information	2.3 (SD: 1.1)
**Executive**	7. Scheduling Work and Activities	2.6 (SD: 1.1)
	8. Resolving Conflicts	3.0 (SD:1.3)
	9. Negotiating with Others	2.7 (SD: 1.2)
	10. Coordinating Work and Activities	2.3 (SD: 1.2)
	11. Guiding, Directing, and Motivating Subordinates	2.5 (SD: 1.2)
	12. Developing Objectives and Strategies	2.6 (SD: 1.2)

Note: Range: 1–5 (1 = low; 5 = high)

## Results

### Descriptive analyses

The mean age of the sample under investigation was 49 years (SD 9.3). The majority of participants were male (58%), had a low or middle educational level (59%), and was working full-time (85%).

Compared to the sample under analysis, individuals whose questionnaires were excluded from analyses according to the aforementioned criteria (see Fig. 1) did not differ in terms of gender (x^2^ = 1.734, df = 1, p = .188), but were more educated (x^2^ = 42.865, df = 2, p < .001) and obviously older (z =-30.532, p < .001) as age was restricted a certain age range.

### Mental demands at work

A summary of the ratings of perceived stress related to mental demands can be found in Table 2. The overall mean of perceived stress was 2.4 (SD: 0.9) with respect to verbal mental demands and 2.6 (SD: 0.9) with respect to all executive demands. The executive demand „Resolving conflicts“ was perceived as specifically stressful (mean: 3.0), whereas „Coordinating work and activities (executive demand)“, as well as „Getting Information“, „Evaluating information“ and „Analyzing Information (verbal mental demands)“ were perceived as least stressful (mean: 2.3).

### Main effects

Results of the unconditional effects adjusted for age, gender, education, income, and occupation and weighed for age, gender and level of education can be found in Table 3. With regard to perceived stress of mental demands, verbal demands were significantly related to chronic stress (β = 0.145, p = .021) and work overload (β = 0.192, p = .031), as one unit change in perceived stress of verbal demands is associated with an increase by 0.145 (chronic stress) and 0.192 (work overload). With regard to the second parameter, greater perceived stressfulness of executive demands, were significantly associated with more chronic stress (β = 0.288, p < .001), higher work overload (β = 0.271, p < .001) and higher work discontent (β = 0.164, p = .003). The same applies to resilience, which was significantly with all three stress domains. In other words, higher levels of resilience was linked to lower levels of job discontent (β = -0.019, p = .001), chronic stress (β = -0.025, p < .001)and work overload (β = -0.019, p = .009). In summary, it was possible to predict 26.3% of chronic stress score variance, 20.0% of work overload score variance and 13,5% of work discontent score variance. No signicant associations were found between the covariates and the three outcome variables except for employment status, as working part-time was associated with higher chronic stress and work overload (see Tabel 3).

**Table 3:** Unconditional effects of perceived stress related to mental demands and resilience on objective stress and work-related dimensions weighed for age, gender and level of education

	Model 1: Chronic Stress		Model 2: Work Overload		Model 3: Work Discontent	
	β, 95% CI	p	β, 95% CI	p	β, 95% CI	p
Resilience	**-0.025 (-0.036; -0.013)**	**< 0.001**	**-0.019 (-0.338; 0.005)**	**0.009**	**-0.019 (-0.030; -0.007)**	**0.001**
Verbal MD	**0.145 (0.022; 0.267)**	**0.021**	**0.192 (0.018; 0.367)**	**0.031**	-0.007 (-0.144; 0.130)	0.922
Executive MD	**0.288 (0.179; 0.397)**	**< 0.001**	**0.271 (0.138; 0.405)**	**< 0.001**	**0.164 (0.055; 0.272)**	**0.003**
Gender	0.124(-0.083; 0.332)	0.241	-0.061 (-0.379; 0.258)	0.708	-0.014 (-0.227; 0.198)	0.896
Age	0.001 (-0.009; 0.010)	0.898	− 0.002 (-0.087; 0.042)	0.494	-0.001 (-0.011; 0.011)	0.983
Education	0.046 (-0.109; 0.201)	0.563	0.034 (-0.178; 0.246)	0.755	0.076 (-0.107; 0.260)	0.415
Income	-0.020 (-0.103; 0.063)	0.639	0.026 (-0.090; 0.142)	0.663	-0.070 (-0.151; 0.011)	0.090
Employment status	**0.372 (0.101; 0.643)**	**0.007**	**0.501 (0.125; 0.877)**	**0.009**	-0.156 (-0.431; 0.118)	0.265
Axiety	-0.041 (-0.419; 0.338)	0.834	-0.129 (-0.561; 0.302)	0.557	0.052 (-0.360; 0.464)	0.804
Depression	-0.019 (-0.235; 0.197)	0.4863	-0.023 (-0.423; 0.376)	0.909	0.226 (-0.109; 0.560)	0.186
R^2^	0.263		0.200		0.135	

Note: MD = mental demands; CI = confidence interval; covariates: gender (ref. male), age, education, income, employment status (ref. full-time), diagnosis of anxiety disorder (ref. no), diagnosis of depression (ref. no); weighed for age, gender and level of education to be representative of the German general population

### Interaction effects

Regarding the moderation analyses, no significant interaction effects could be obtained with regard to executive mental demands and the three occupational outcome variables (see Table 4). Interaction analysis was only relevant with regard to work overload, indicating that the interaction between verbal mental demands and resilience significantly affects work overload (β = 0.021, p = .021). In addition and in order to back up these results, margin plots based on the distribution of resilience confirmed that the effect of resilience depends on the degree of verbal mental demands.

**Table 4:** Conditional effects of perceived stress related to mental demands and resilience on work-related stress weighed for age, gender and level of education

Overall	Model 1: Chronic Stress		Model 2: Work Overload		Model 3: Work Discontent	
	β, 95% CI	p	β, 95% CI	p	β, 95% CI	p
Resilience	**-0.234 (-0.328; -0.014)**	**< 0.001**	**-0.018 (-0.031; -0.006)**	**0.005**	**-0.018 (-0.028; -0.008)**	**0.001**
Verbal MD	**0.158 (0.038; 0.278)**	**0.010**	**0.213 (0.047; 0.379)**	**0.012**	0.003 (-0.138; 0.144)	0.968
Executive MD	**0.283 (0.175; 0.390)**	**< 0.001**	**0.251 (0.125; 0.377)**	**< 0.001**	**0.161 (0.048; 0.275)**	**0.005**
Resilience x Verbal MD	0.012 (-0.001; 0.026)	0.079	**0.021 (0.003; 0.040)**	**0.021**	0.006 (-0.009; 0.022)	0.440
Resilience x Executive MD	0.002 (-0.013; 0.010)	0.803	-0.015 (-0.030; 0.001)	0.052	0.002 (-0.013; 0.017)	0.774
Gender	0.100 (-0.108; 0.309)	0.344	-0.101 (-0.423; 0.221)	0.538	-0.033 (-0.248; 0.183)	0.766
Age	-0.001 (-0.010; 0.010)	0.980	-0.001 (-0.015; 0.015)	0.986	-0.001 (-0.011; 0.010)	0.898
Education	0.057 (-0.097; 0.211)	0.471	0.043 (-0.166; 0.252)	0.689	0.089 (-0.095; 0.273)	0.344
Income	-0.029 (-0.112; 0.053)	0.488	0.022 (-0.096; 0.140)	0.719	-0.080 (-0.159; 0.001)	0.051
Employment status	**0.345 (0.086; 0.605)**	**0.009**	**0.498 (0.134; 0.862)**	**0.007**	-0.185 (-0.450; 0.080)	0.171
Anxiety	0.100 (-0.342; 0.542)	0.657	0.002 (-0.460; 0.465)	0.992	0.127 (-0.310; 0.565)	0.569
Depression	0.031 (-0.202; 0.264)	0.795	-0.014 (-0.415; 0.387)	0.946	0.276 (-0.072; 0.625)	0.120
R^2^	0.285		0.184		0.145	

Note: MD = mental demands; CI = confidence interval; covariates: gender (ref. male), age, education, income, employment status (ref. full-time), diagnosis of anxiety disorder (ref. no), diagnosis of depression (ref. no); weighed for age, gender and level of education to be representative of the German general population

## Discussion

The aim of this study was to build on previous research on occupational mental demands and stress and investigate the link between these demands and occupational well-being in a broad sample and across a variety of occupations. Secondly, we aimed at exploring a possible moderating effect exhibited by the levels of resilience, as resilience may influence how stressful an individual perceives certain mental demands by serving as a personal resource against distress [31]. According to the theoretical framework put forth by the Job Demands Resources Model [32], job demands, such overload, have been shown to foster the motivational process and produce favorable results (such as job satisfaction and performance, as well as work engagement) [33]. However, when workload demands outweigh available resources, it leads to a process of health impairment that has unfavorable effects (such as stress and burnout) [32, 34].

In the current study including a wide range of occupations, the mental demand „Resolving conflicts“ was perceived as the most stressful demand, similar to another study with a smaller and non-representative sample [19]. Therefore, this finding seems to apply to many profession types or occupational groups.

The first hypothesis, that perceived stress of mental demands may increase chronic stress, work overload and work discontent was confirmed partly, as results show that higher perceived stress of executive mental demands was significantly associated with higher levels of chronic stress, work overload and job discontent, whereas verbal demands were signifcantly related to chronic stress and work overload. The current status of knowledge implies that neurochemical processes seem to be relevant in explaining why mental demands may be protective against cognitive decline [35]. However, the current study underlines that mental demands may also accelerate negative effects, such as stress and workload. The question remains whether cortisol-levels that are known to be frequently altered in response to chronic or re-occurring stress also explain the current findings [36]. Physiological explanation such as increases in cortisol or changes on the hypocortioid axes might explain why high mental demands are associated with increases in stress. Future research should therefore focus on the endocrinological processes that may explain the current finding that perceived stress of mental demands increases work overload, chronic stress and discontent. Otherwise, high levels of workload, stress and discontent may not only give grounds to mental health issues [37, 38] but also foster turnover, early retirement and absenteeism among employees [39, 40].

The second hypothesis could not completely be confirmed by the current study sample, as interaction analyses did only reveal some significant interaction effects by resilience. In other words, only the interaction between resilience and perceived stress associated with verbal mental demands revealed a significant effects when investigating the relationships between mental demands, work overload and resilience. No significant interaction effects were found regarding perceived stressfulness of executive mental demands. Furthermore, the results indicate that even if less resilience and less verbal demands are accompanied by increasing overload, resilience may only exert preventive functioning (i.e. reduce work overload) up to a certain point. The amount of stress, that persists through mental demands at the workplace seems to be robust and hardly be affected by these personal resources. In the current study, the protective function of resilience was therefore rather limited. In general, compared to excecutive mental demand, verbal demands are more likely to depend on crystallized intelligence [11] and may therefore be less susceptible by individual and dynamic resources, such as resilience, even if there is evidence that resilience may be a positive resource for individuals working at stressful workplaces [41]. In this context, studies suggest that high levels of resilience are associated with lower risk for burnout and psychosocial health [42, 43]. Therefore, results of the current study were rather surprising, especially as the mean resilience score in this sample was high and comparable to a study including a representative sample of the general public [29]. According to the systematic self-reflection model of resilience strengthening, the way individuals cope and react with psychological stressors shapes their capacity for resilience so that resilience may not act on stress or stress reactions, but rather be affected by how individuals deal with certain psychological stressors [44]. In other words, how mental demands are perceived in terms of stressfulness may influence the protective function of resilience and therefore influence chronic stress, work overload and discontent. Therefore, the association between mental demands and resilience still needs further attention in order to transfer this knowledge into practice and to determine specific cut-offs or ranges of mental demands that foster or drain the positive effects of resilience.

Future studies could also investigate this topic using specific work resilience scales. It might be possible that resilience in general may not have an impact on how people deal with stress due to mental demands. Rather, specific characteristics of (occupational) resilience might be of greater importance. In this context, it could also be investigated how work engagament may alter perceived stress of mental demands. Work engagament has been linked to several stress-biomarkers (i.e. cortisol levels or activity on the hypothalamic-pituitary-adrenal axis) as well as burnout and work overload [36]. Similarily, other inter-individual factors that determine how perceptions of stress associated with mental demands may play a role – such as the use of different coping styles [45]. In other words, individual coping mechanisms might explain why demands may have positive effects for some people, but results in more distress and overload in others. Perceived stress of mental demands may change on a day to day basis. In other words, there are not only inter-individuals factors, but also intra-individuals factors such as physical (i.e. overall health or fatigue) or mental well-being (i.e. depressiveness or anxiety, especially work anxiety) or the possibility to (sufficiently) recover from work that determine a person’s appraisal of stress. Experimental studies could be used to determine these parameters in order to develop individual interventions that help to reduce negative impact of perceived stress of mental demands, without eliminating their positive effects on cognitive decline as previously reported [8, 9, 14].

The underlying data was taken form a large cohort sample representative of the German public and is the first study investigating the link between resilience and occupational mental demands. Unfortunatley, no power-analysis has been conducted before, therefore, it may be possible that the statistical power needed to find significant interaction effects is not sufficient. Data was collected during the COVID-19 pandemic which may have biased the results on stress as studies find higher stress levels associated with the mandemic, especially at the workplace of individuals [33]. Furthermore, during the last years, the structural dimensions of work have changed, i.e. having the possibility to work in remote (fully or in part). This may also lead to changes in mental demands and how they are perceived as being stressful. Therefore, more research is needed to investigate this not only by comparing different groups, but also intraindividually in order to detect changes caused by the establishment of remote work using pre-post-comparisions. In addition, this may also be related to distribution of work types or occupations (such as service jobs vs. office jobs, having many social relationships at work or none), as this may contribute to differences in mental demands and perceived stress. The underlying, cross-sectional data is based on self-reports. Therefore, studies including more objective measures (i.e. physiological stress measurements), and data collection at different time-points may extend our findings and further contribute to a better understanding of the association between mental demands and stress at work.

## Conclusion

Mental demands at work have been shown to be protective against cognitive decline in old age. However, the current study shows that stressfulness of mental demands perceived as high can be associated with higher chronic stress, work overload and work discontent. Therefore, mental demands should be targeted by occupational interventions that aim to improve job conditions and employees‘ overall well-being. In future, further individual solutions must be found in order to determine to what extent mental (excessive) demands can be reduced. In other words, person and job should be merged in such a way that the mental demands match the person’s abilities and thus bring about the benefits already demonstrated, at the same time that overwork or mental load should not lead to stress that manifests itself, leading to others negative health effects. Resilience did only partly buffer negative work-related effects regarding the association between verbal mental demands and work overload. Future research should focus on further personal resources that may be helpful in reducing the negative impacts of stressful mental demands at work besides resilience.

## Data Availability

The data that support the findings of this study are available from the corresponding author upon reasonable request.
